# Volume Nine

**Published:** 1870-07

**Authors:** 


### Editorial Department.

### VOLUME NINE.

Thus closes volume nine. Thanks to a liberal profession we have been sustained with generous and satisfactory support. The past indulgence and favor of the profession encourage the highest expectations for the future, and at no period of our history could we commence a new volume with such positive assurauces of success. It is matter of personal satisfaction and pride that nine years of uninterrupted progress in all the essentials of true prosperity should already have attended the Journal, and that though surrounded by numerous and attractive rivals, it still continues to increase in strength and influence. We have reason for thankfulness that an enterprise in which we have been so deeply interested should have been so successful; but not unto us, but to our contributors and supporters be all the praise.

For the future we pledge our best efforts, warmly thanking our friends for their numerous favors, earnestly hoping they may continue to kindly remember us in the future as they have liberally done in the past, we commence volume ten.

